# Gap junctional communication in the intestine affects social feeding behavior via FLP-21 and the NPR-1 mediated signaling pathway

**DOI:** 10.17912/micropub.biology.002160

**Published:** 2026-06-02

**Authors:** Lisa Calabro, Maureen Peters

**Affiliations:** 1 Science Communications, IQ Solutions, Inc, Rockville, MD, USA; 2 Biology, Oberlin College

## Abstract

Communication between the gut and the nervous system coordinates aspects of behavior and responses to stressors.&nbsp; When actively feeding, *C. elegans* executes a digestive motor program triggered by a calcium wave that requires gap junctional connections along the intestinal length.&nbsp; Loss of function of the innexin-16 ( *inx-16* ) gap junctional subunit results in altered group feeding behavior, leading to social feeding characterized by increased body contact, or clumping, between animals. Using genetic analysis, our data shows that *inx-16* genetically interacts with *npr-1* and *flp-21 ,* but not *daf-7* . Thus, gut-to-brain signaling influences social versus solitary feeding behavioral choice.

**Figure 1. inx-16 mutation results in enhanced aggregation and genetically interacts with both npr-1 and flp-21:** **Figure 1** : *inx-16* loss of function increases aggregation, and genetic interaction assays show an interaction between *flp-21* and *npr-1* but not *daf-7* . (A) Aggregation fractions for N2 ; *npr-1 ( ad609 )* ; *inx-16 ( tm1589 )* mutant with inx-16 (+) expressed in the muscle (mus.) and *inx-16* mutant with inx-16 (+) expressed in the adult intestinal (int.), both labeled as + “rescue”, n=9 for all strains. (B) Aggregation fractions for defecation mutants compared to N2 , mutants used: *aex-2 ( sa3 )* ; *exp-1 ( sa6 );* *pbo-4 ( ok583 )* ; *pbo-5 ( ox4 );* n=6 for all strains *.* (C) Aggregation fractions for single and double mutants of the following genes: *inx-16 ( tm1589 ); npr-1 ( ad609* ); *daf-7 ( e1372 );* n=6 for all strains. (D) Aggregation fractions for single and double mutants of the following genes: *flp-21 ( ok889 ); inx-16 ( tm1589 )* alongside N2 ; n=8 for all strains. Statistical significance: *p<0.05; **= p<0.01; ***<0.001; ****<0.0001; ns is not significant. Brackets used for comparisons between two mutants; line used across three strains for genetic interactions.

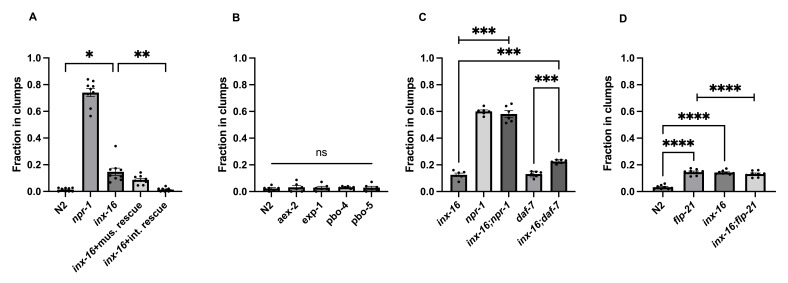



## Description

Organisms adapt to their dynamic internal and external environments by leveraging systemic physiological responses using signaling pathways involving multiple tissues and extensive cross-talk. Recently, the gut has emerged as an important signaling center keenly attuned to an animal's feeding state, food quality, and pathogenic exposure.&nbsp;Within *C. elegans* , gut-derived secreted peptide signals influence feeding, stress response, waste release and avoidance behavior by acting on neurons of the enteric and central nervous systems (Artan et al., 2016; Jia et al., 2024; Sural et al., 2025). For example, INS-7 and INS-35 regulate the NSM neuron to adjust pharyngeal pumping rates (Sural et al., 2025).&nbsp; Additional insulin family members such as INS-3 and INS-11 function in gut-to-brain signaling pathways (Lee and Mylonakis, 2017; Veuthey et al., 2025).&nbsp; Several non-insulin peptides are also involved.&nbsp; FLP-2 (FMRFamide-like peptide) acts via the interneuron AIY to induce responses to oxidative stress within the animal (Jia et al., 2024). NLP-40 (Neuropeptide-like protein) is released to instruct the posterior enteric neurons, AVL and DVB, to elicit steps of the digestive/defecation motor program; its release is tied to a periodic calcium wave that occurs in well-fed animals with a regular frequency of 45-55 seconds (Choi et al., 2023; Mahoney et al., 2008; Wang et al., 2013).

This regular calcium wave may trigger release of additional peptides that influence nervous system behavior more broadly. Several mutants with poor digestive waste release originally identified due to their constipated phenotypes turn out to encode integral components of peptide processing and release such as *egl-3* , *egl-21* , *aex-1* , *aex-4* , and *aex-5* (Husson et al., 2007; Khawand et al., 2026; Mahoney et al., 2008).&nbsp;At least one peptide, NLP-40 , is released in time with the digestive motor program, via activation of synaptotagmin-2 ( *snt-2 )* , a calcium-sensing protein that triggers DCV fusion (Wang et al., 2013). The tyramine response pathway offers additional evidence of calcium-triggered peptide release (Veuthey et al., 2025). This enticing evidence suggests that the rhythmic calcium flux that triggers NLP-40 release, and other steps of the digestive/defecation motor program, may be used more broadly to influence behavioral choices where feeding and nutritional status are relevant. With this notion in mind, we explored an observation of social feeding behavior in a mutant that alters intestinal calcium wave dynamics.

The *innexin-16* ( *inx-16* ) gap junction subunit is critical for periodic intestinal calcium waves associated with the digestive motor program; loss of function *inx-16* mutants disrupt the intestinal calcium wave and greatly reduce the digestive motor program steps that require NLP-40 release (Peters et al., 2007). During routine maintenance of *inx-16 ( tm1589 )* , we observed increased aggregation of well-fed worms and became intrigued. To explore this further, we assayed for social feeding behavior quantitatively. For comparison, we included N2 , a solitary strain, and a social feeding strain, *npr-1 ( ad609 ).* The gene, *npr-1* , encodes a *C. elegans* receptor related to neuropeptide Y and is neuronally expressed. When active, NPR-1 inhibits the tendency of animals to aggregate in groups, suppresses oxygen level preferences, and affects bordering (de Bono and Bargmann, 1998; Gray et al., 2004). The *npr-1* allele we used, *ad609* , has been characterized to be a strong loss of function allele, equivalent to a genetic null (de Bono and Bargmann, 1998). Our results show that *inx-16* mutant animals maintain contact with other animals more frequently than wild-type ( N2 ) solitary worms; young adult *inx-16 ( tm1589 )* animals demonstrated a significant difference in the number of *inx-16* animals found in direct contact with their peers compared to N2 (~0% vs. ~15%; Figure 1A ). Yet, the *inx-16* phenotype is much weaker than the *npr-1 ( ad609 )* allele (~80% vs. ~15%; Figure 1A ) *.*

To verify that INX-16 functions within the intestine to cause the observed tendency to aggregate, we employed tissue specific rescue of *inx-16* mutants. Our and others' *inx-16* expression analyses indicated that *inx-16* was only expressed in the intestine, yet two studies have provided phenotypic evidence suggesting additional areas of function (Bhattacharya et al., 2019; Liu et al., 2013; Sojka et al., 2025).&nbsp;Intestine-specific rescue was achieved using a construct with a short, well-defined fragment, regulatory sequence of the egg yolk protein, vitellogenin-2, ( *vit-2* ) gene, placed upstream of an INX-16 translational fusion (MacMorris et al., 1992). Prior work using this construct led to full rescue of the defecation defects in adults (Peters et al., 2007).&nbsp;Likewise, solitary feeding behavior was restored in the *inx-16* + *p vit-2* intestinal rescue line ( Figure 1A ). By contrast, driving expression of *inx-16* in body wall muscles, using the *myo-3* promoter, was unable to restore solitary (wild-type) feeding behavior ( Figure 1A ; Liu et al., 2013). These data suggest that intestinal INX-16 function contributes to the animal's feeding behaviors.

Having shown that *inx-16 '* s actions in the intestine can rescue the social feeding phenotype, we queried whether aggregated feeding was a common feature of mutants with impaired digestive waste release (Thomas, 1990). Defecation mutants induce constipation. One constipated mutant with defects in the expulsion step, *exp-1* , disrupts an excitatory GABA-ergic receptor in enteric muscles and has been shown to mildly increase social feeding behavior (Beg and Jorgensen, 2003; Bendesky et al., 2012). We assayed this mutant, and others, selecting a set of mutants that affect each defecation motor step and all tissue types involved in the motor program (Branicky and Hekimi, 2006; Thomas, 1990). Two genes required for the first motor step (the posterior body contraction), the intestinal sodium proton exchanger, *pbo-4 /nhx-7* , and the downstream proton-gated ion channel, *pbo-5* , localized to body wall muscles, were tested (Beg et al., 2008; Pfeiffer et al., 2008). Neither of these Pbo mutants showed increases in social feeding in comparison to N2 ( Figure 1B ). The later steps of the program, anterior body contraction and enteric muscle contraction, require NLP-40 release from the intestine to stimulate GABA release from enteric neurons, AVL and DVB (Wang et al., 2013).&nbsp; NLP-40 binding activates AEX-2 , a g-protein coupled receptor, on these neurons (Allman et al., 2016; Mahoney et al., 2008). Like the Pbo mutants, *aex-2 ( sa3 )* showed no significant increase over wild-type in our aggregation assay ( Figure 1B ). In our assays, *exp-1 ( sa6 )* did not increase aggregation, which differs from prior findings that reported a small but significant increase (Bendesky et al., 2012). Though our defecation mutant panel is not exhaustive, these data suggest that the social feeding phenotype of *inx-16* is not broadly shared with mutants suffering from constipation; disrupting defecation per se does not alter social feeding behavior in *C. elegans* .

Two key gene pathways control the choice between solitary and social feeding behavior: *npr-1* and *daf-7* . Therefore, we used genetic assays to elucidate potential connections between *inx-16* and these known pathways (de Bono and Bargmann, 1998; de Bono et al., 2002). Double mutants of *inx-16* with *daf-7 ( e1372 )* and *npr-1 ( ad609 )* were constructed and tested alongside single mutants to determine whether the double mutant lines had enhanced aggregation.&nbsp;The combination of *inx-16* and *daf-7 ( e1372 )* enhanced aggregation substantially, with the *inx-16 ; daf-7* double mutant exhibiting increased aggregation compared to the *daf-7* mutant alone, approximately 23% for the double compared to ~13% for either single mutants ( Figure 1C ). This additive effect is consistent with *inx-16* functioning at least partly independently of *daf-7* . By contrast, the combination of *inx-16* and the strong loss of function *npr-1* allele, *ad609* , did not augment the social feeding level relative to that of *npr-1* alone. The *inx-16 ; npr-1* double mutants exhibit no increase in aggregation compared to the *npr-1* mutant alone (60% for the double compared to 58% for the single mutant, Figure 1C ). The results of a two-way ANOVA analysis support a highly significant genetic interaction between *inx-16* and *npr-1* (P < 0.003; F = (1, 20)=19). These findings are consistent with *inx-16* functioning via the *npr-1* pathway to mediate social feeding behavior.

Since our findings placed *inx-16* within the *npr-1* pathway rather than the *daf-7* pathway, we searched for NPR-1 ligands with evidence of intestinal expression. Of the two identified NPR-1 ligands, FLP-18 and FLP-21 , *flp-21* showed intestinal expression in addition to a subset of neurons (Kubiak et al., 2003; Rogers et al., 2003). The *flp-21* mutation causes a significantly increased propensity for animals to aggregate, enhancing aggregation to ~15% ( Figure 1D ). Interestingly, *flp-21 's* magnitude of impact on feeding behavior is similar to that of *inx-16* mutation, ~15% for *flp-21* vs. ~14% for *inx-16* ( Figure 1D ; Rogers et al., 2003). Thus, we hypothesized that FLP-21 could be released from the intestine following calcium waves in a manner analogous to NLP-40 release. To begin to investigate this model, we explored epistasis between *inx-16* and *flp-21* . The double mutant line exhibited an aggregation profile very similar to that of either individual mutant strain ( *inx-16 ; flp-2 1 = ~* 13%; Figure 1D ). Statistical analysis for gene interaction upholds this epistatic relationship (P < 0.0001; F = (1, 28) = 89). Our results support a model placing FLP-21 release downstream of INX-16 function, and by inference the rhythmic intestinal calcium wave that directs the digestive motor program (Dal Santo et al., 1999; Espelt et al., 2005; Teramoto and Iwasaki, 2006).&nbsp;

Due to *flp-21* 's reported intestinal localization, we suggest that *inx-16 's* social feeding phenotype may be due to reduced FLP-21 release from the intestine. Since NPR-1 signaling dampens social feeding, loss of FLP-21 would increase aggregation, as seen in both *inx-16* and *flp-21* mutants. Our model is supported by the previously reported epistasis analysis of *flp-21 ( pk1601* ) and *npr-1 ( ad609 );* this *flp-21* allele did not enhance *npr-1* ( *ad609* )'s social feeding level, matching our results ( Figure 1C ; Rogers et al., 2003). Given that *flp-18* , the other known NPR-1 ligand, is not expressed in the intestine, *flp-18* presumably remains active in the *inx-16* mutant, allowing ample residual suppression of social feeding to result in the modest aggregation seen in both the *inx-16* and *flp-21* mutants ( Figure 1D ; Kapahi et al., 2010).

In summary, our findings demonstrate a behavioral feeding phenotype associated with disrupted intestinal calcium wave dynamics. We propose that this phenotype is due to disruption of FLP-21 mediated activation of NPR-1 . Moreover, the social feeding phenotype of *inx-16* suggests that the periodic intestinal calcium waves serve to regulate peptide release broadly, influencing a host of behaviors beyond defecation and adding to the growing body of evidence illustrating the importance of gut-to-brain signaling for animal behavior.&nbsp;

## Methods

**Strain maintenance and construction**

*C. elegans* strains were cultured on standard NGM plates seeded with OP50 bacteria using standard protocols. Double *daf-7* and *npr-1* mutant strains were generated using standard genetic crossing, selection based on dauer or aggregation phenotype, followed by sequencing to confirm genotypes. For *flp-21* , the large deletion allowed the use of PCR tracking prior to sequencing.

**Social feeding assay**

Young adult animals were assayed blindly using a protocol adapted from De Bono and Bargmann (1998). Strains containing the *daf-7 ( e1372 )* mutation were reared at 15 ^o^ C to prevent dauer formation. NGM plates were seeded with 200 mL of OP50 in a circular lawn about 25 mm in diameter. Approximately 150 well-fed day 1 adult worms were added and left at 20-22 ^o^ C for 2 hours. Then, the fraction of animals in contact with two or more other animals along at least 50% of their body length was tallied by visual inspection. For each panel's experiments, the full set of strains was tested each assay day and the full set of assays were completed in a short time period.

**Statistical analysis and graphing**

For statistical analysis, Graphpad/Prism (Version 11 for macOS) was used. Each panel's data set was tested for Gaussian distribution. Only one panel's data set (A) showed some non-Gaussian distribution, so Kruskal-Wallis program was applied. Otherwise, ANOVA analysis followed by multiple comparisons using Tukey's test (one-way ANOVA for panels A and B; two-way ANOVA for panels C and D) was used. Statistical deviation was considered significant if p<0.05. Bar graphs show all data points, mean and standard error of the mean. p value scores are listed for informative comparisons relevant to our study. Differences between *npr-1* and N2 , or other mutants that were previously published and/or strikingly obvious were not specifically noted. For example, statistical tests between N2 and *npr-1 ; inx-16* and *npr-1 ; npr-1* and *daf-7* were not highlighted to simplify the figures.

## Reagents

**Table:** 

**Strain**	**Genotype**	**Available from**
N2	Bristol isolate	CGC
EG3528	*inx-16 ( tm1589 ) I *	National BioResource Project (NBRP)
TA145	*inx-16 ( tm1589 ) I; oxEx559 (p vit-2 : INX-16 :GFP) *	
CB1372	*daf-7 ( e1372 )* III	CGC
DA609	*npr-1 ( ad609 )* X	CGC
RB982	*flp-21 ( ok889 )* V	CGC
RB793	*pbo-4 ( ok583 )* X	CGC
EG4	*pbo-5 ( ox4 )* V	CGC
JT3	*aex-2 ( sa3 )* X	CGC
JT6	*exp-1 ( sa6 )* II	CGC
TA149	*inx-16 ( tm1589* )I; *npr-1 ( ad609 )* X	
TA155	*inx-16 ( tm1589* )I; *daf-7* ( *e1372* )III	
TA156	*inx-16 ( tm1589* )I; *flp-21 ( ok889 )* V	
ZW701	*inx-16 ( tm1589 ) I; zwEx701[p myo-3 :: inx-16 (+)::GFP + p myo-3 ::GFP] *	Zhao-Wen Wang Lab
